# Modification of Glass/Polyester Laminates with Flame Retardants

**DOI:** 10.3390/ma14247901

**Published:** 2021-12-20

**Authors:** Adriana Dowbysz, Mariola Samsonowicz, Bożena Kukfisz

**Affiliations:** 1 Department of Chemistry, Biology and Biotechnology, Bialystok University of Technology, Wiejska 45A Street, 15-351 Bialystok, Poland; m.samsonowicz@pb.edu.pl; 2 The Main School of Fire Service, Faculty of Security Engineering and Civil Protection, Slowackiego Street 52/54, 01-629 Warsaw, Poland

**Keywords:** glass/polyester laminates, flammability, flame retardants, unsaturated polyester resin

## Abstract

This paper presents a review of flame retardants used for glass/polyester laminates. It concerns flame retardants withdrawn from use such as compounds containing halogen atoms and flame retardants currently used in the industry, such as inorganic hydroxides, phosphorus and nitrogen-containing compounds, antimony, and boron compounds, as well as tin–zinc compounds. Attention is also drawn to the use of nanoclays and the production of nanocomposites, intumescent flame retardant systems, and mats, as well as polyhedral oligomeric silsesquioxanes. The paper discusses the action mechanism of particular flame retardants and presents their advantages and disadvantages.

## 1. Introduction

Numerous advantages of plastics such as low weight, hardness, resistance to chemicals, water and impact, cheapness, strength, durability, and good electrical properties encourage their use in almost all industrial fields [1]. The packaging and automotive industry, as well as building and construction, comprise three branches accounting for nearly 70% of all plastics used in Europe, while the latter accounts for 20% [2]. The most frequently used polymers in that branch are polyethylene (PE), polyvinyl chloride (PVC), polymethyl methacrylate (PMMA), polystyrene (PS), polypropylene (PP), and different types of resins such as phenol-formaldehyde (PF), organic silicon (OSR), or polyester resin (PR) [3]. The global production and processing of resins have both significantly increased over the past few decades. Approximately, a 1182% growth was observed from the 1950s to 2018 [4]. Even the COVID-19 pandemic and subsequent economic fallout did not stop the later growth of plastic resin production. In fact, only this segment among chemical sectors experienced post-COVID-19 positive growth in 2020 [5].

However, because of their high fuel values, polymers and their composites, widely used in furniture, fabrics, automotive parts, and for some equipment in housing, may cause danger. Fire safety requirements are described in detail for several plastics applications. When particular materials do not achieve a proper fire standard, there is a necessity to use flame retardants [6]. 

Flammable materials, including polymers, used in various areas of human life pose a high fire hazard. Flame retardants reduce that risk and help achieve suitable levels of fire protection. These substances have been used for many years and each has its advantages and disadvantages. Their effectiveness in reducing the flammability of different materials depends on many factors: (i) the type of the material, its application, and the environment in which it will be used; (ii) the compatibility of a flame retardant with the specific material; and (iii) processing techniques. Some flame retardants release toxic gases when burned and some can cause the early degradation of certain materials. Some of them are hygroscopic and may cause the hydrolysis of the product. Some fire retardants must be used in large quantities in order to be effective, which in turn causes the deterioration of mechanical properties and decreases the interfacial adhesion between different ingredients of the composite.

Currently, there is an increased interest in flame retardants safe for people and the environment, which, at the same time, do not adversely affect material properties. The aim of this study was to review various types of flame retardants used to reduce the flammability of glass/polyester laminates. The main focus was on assessing the advantages and disadvantages of these agents and on the synergy of their actions. Particular attention was paid to their impact on the environment. 

### 1.1. Characterization of Glass Fiber Reinforced Polyesters

Polymer matrix composites may be divided into Glass Fiber Reinforced Polyester (GFRP), Hybrid Fiber Reinforced Polyester (HFRP), and Natural Fiber Reinforced Polyester (NFRP). They are commonly used in many applications replacing metals [7]. Since the 1940s, GFRPs began to develop rapidly due to their fabrication adaptiveness, durability, and low price [8].

GFRP’s matrix consists of a polymer resin reinforced by glass fibers. Widely used unsaturated polyester resins consist of linear polycondensation products, such as unsaturated and saturated acids or their anhydrides, diols or oxides, and vinyl monomers. They may be divided into ortho-resins, iso-resins, bisphenol-A fumarates, chlorendics, and vinyl ester resins. The general purpose ortho-resins are based on phthalic acid, maleic anhydride or fumaric acid, and glycols. Their limitations are reduced thermal and chemical resistance and processability, but they are less crystalline and more compatible with styrene. Iso-resins are based on isophthalic acid, maleic anhydride or fumaric acid, and glycol. They are more expensive than ortho-resins. This is due to their better thermal and chemical resistance and improved mechanical properties. Higher hardness and better thermal performance of bisphenol-A fumarates are observed when bisphenol-A is built in the backbone. Chlorine or bromine-containing anhydrides or phenols are used when the improvement of flame retardancy is needed. Vinyl ester resins are bisacryloxy and bismethacryloxy derivatives of epoxy resins. They are made from acrylic acid or methacrylic acid with epoxy resin. Low styrene emission, high tensile strength, heat deflection temperature, and corrosion resistance are their prime advantages [9].

Crosslinking reactions of unsaturated polyester resin (UPR) take place at room temperature after the addition of an initiator such as methyl ethyl ketone, and an accelerator such as cobalt(II) naphthenate. This process is based on the copolymerization of UPR and an unsaturated monomer, which is mostly styrene [10]. Four types of crosslinking reactions take place: inter or intramolecular crosslinking with or without linking through styrene monomers, branching polyester by styrene, and styrene homopolymerization [11].

Glass fibers come in many forms, including woven roving, chopped strand mat, or cloth. Their good wettability results in the formation of strong bonds on the interfacial surface of polymer and glass, and the enhancement of mechanical properties [12]. The fiber length, orientation, and volume fraction are particularly important, as they influence the thermal and electrical conductivities, the density, the mechanical and fire reaction properties, and the cost of the material [13]. Inorganic glass fibers are not flammable. They protect the material from flame and heat, as they can withstand temperatures up to 1100 °C for a considerable time. Inorganic glass fibers delay ignition time. For thermoplastic matrix composites, glass fibers cause the so-called “candlewick effect”, resulting in the faster decomposition of polymer. However, this effect does not occur for the thermoset polymer matrix [14].

The type of glass fiber reinforcement affects the properties of composites. Its classification is shown in Table 1.

Glass/polyester laminates are one of GFRP’s structures, where layers of reinforcement laying over one another are bonded with a matrix [16]. A hand lay-up method is a traditional method for manufacturing laminates, but it is replaced by vacuum bagging or infusion processes [17]. Every method has its limitations, but research shows that vacuum infusion is the most effective [18].

The laminates are often protected with an external layer (gelcoat) made from a resin and high quality fillers, which makes them UV resistant and/or water resistant. It also adds aesthetic value to the final product [19]. These external layers may have a negative effect on flammability properties, as well as slightly increase smoke release [20].

Nowadays, glass/polyester laminates are widely used in the aerospace, electronics, automotive, rail, and sport industries and in the construction industry due to their high resistance to shock, dynamic, and high static loads [21,22]. They are also lightweight, non-magnetic, and corrosion-resistant. However, their disadvantages are high flammability and large toxic smoke production during thermal decomposition [23]. Dense smoke is generated because of the high content of aromatic compounds, such as the styrene and phthalic acid functionalities in the resin, which leads to a reduction in visibility [24].

### 1.2. Characterization of the Combustion Process of Glass Fiber Reinforced Polyesters

Unsaturated polyester resins undergo thermal degradation at temperatures between 350 °C and 410 °C, and their auto-ignition temperature is usually between 420 °C and 440 °C. The limiting oxygen index describing flammability is generally 19–20% (*v*/*v*). The UPR heat of combustion can be up to 40 MJ/kg [25]. For this reason, GFRPs easily ignite when exposed to fire and high temperatures. Besides releasing a lot of heat, smoke, and fumes, a composite exposed to a temperature higher than the glass transition temperature of a polyester matrix can lose its stiffness and strength. Degradation of a GFRP’s mechanical properties occurs during a fire because of thermal degradation and the combustion of the resin. Research shows that an increase in the heat exposure time and heat flux results in the faster deterioration of tension, compression, flexure, and interlaminar shear properties [26]. 

Thereupon, glass/polyester laminates may pose a significant fire hazard. Flame retardancy is not obligatory but can be required in all applications, for example, in underground piping or mining bolts. However, it is crucial for providing fire safety, especially when these composites are used in public transportation or constructions [27]. 

**Figure 1:**
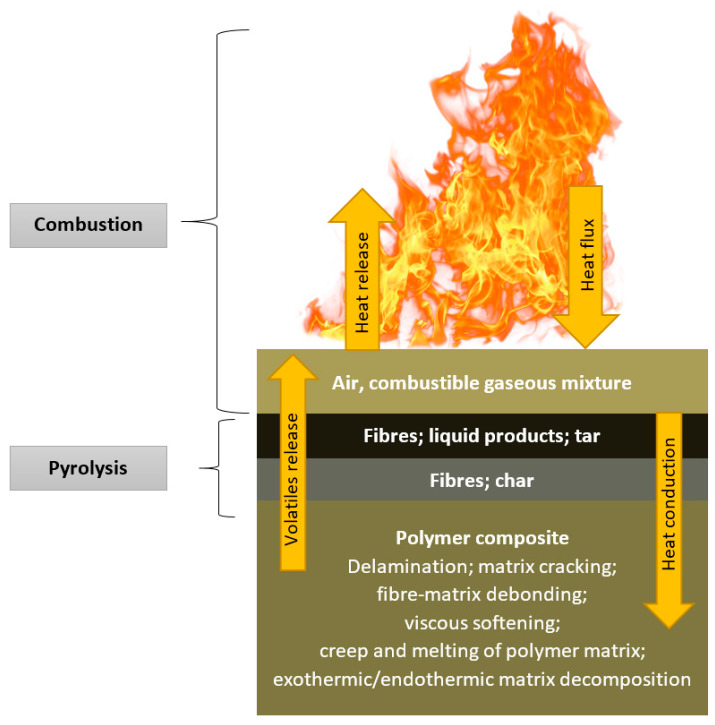
Diagram of the different phases, mechanisms, and reactions taking place during the exposure of the polymer to fire [13].

Thermal decomposition of organic components contained in GRFP starts with releasing volatile gases and soot, leading to the generation of liquid products, tar, and carbonaceous char. Nonflammable volatiles consist of carbon dioxide and water, and the flammable ones include carbon monoxide, methane, and other low molecular weight molecules or short polymer fragments. Diffusion of the latter from the composite into air results in the creation of a combustible gaseous mixture. The ignition of these gases may occur when a reaction with oxygen from air takes place. Products of this reaction comprise carbon dioxide, carbon monoxide, soot, and heat. When released heat exceeds a certain level, new decomposition reactions of organic components take place in the material. This complex phenomenon of the combustion process is shown in Figure 1.

The flammability of GFRP is mainly subordinated to the thermal decomposition of the polymer matrix. The initial scission of polystyrene cross-links results in the formation of free radicals that promote further decomposition and in the emission of volatiles such as carbon monoxide, carbon dioxide, methane, ethylene, propylene, butadiene, naphthalene, benzene, and toluene. The high smoke production rate of GFRP composites also comes from the polymer matrix, particularly from the contained styrene monomer. Another disadvantage is the tendency to lose flaming droplets due to the emission of liquid products and tar during decomposition. 

On the other hand, noncombustible glass fibers also influence the flammability of GFRP. They act as a protective char layer for heat penetrating the material and flammable volatiles flowing out from the material.

When exposed to fire, GFRP can withstand high temperatures, but, at some point, a delamination process may occur, where interlaminar layers mechanically separate. Due to the high growth of fire conditions, hot gases inside the material cannot diffuse outwards, and the pressure builds up, resulting in delamination [13].

## 2. Flame Retardants Used for GFRPs

**Figure 2:**
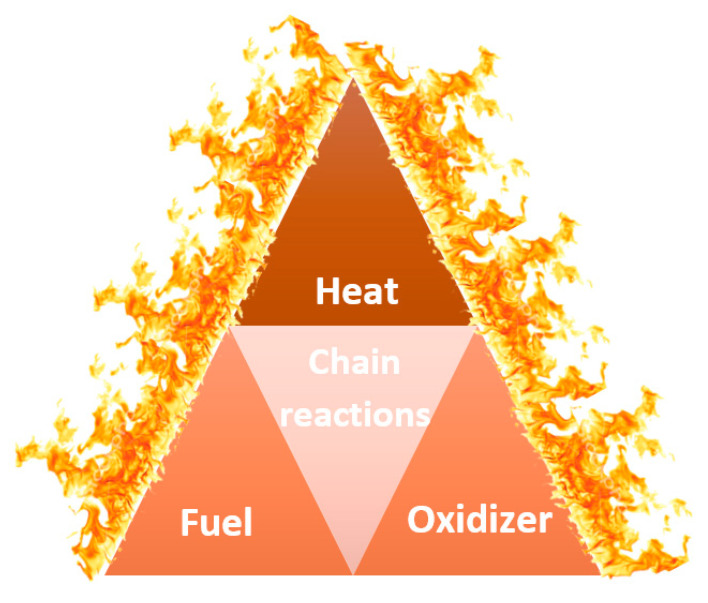
A fire tetrahedron [29].

The combustion process requires the presence of four elements: oxidizer (such as oxygen), fuel, heat, and chain reactions, which form a fire tetrahedron, as presented in Figure 2. Flame retardants affect one or more of those elements to stop the pyrolysis process, shorten the time to ignition, prevent flame spreading, and reduce the amount of released toxic gaseous products [28].

Due to the significant amount of styrene content and the presence of aliphatic chains, UPRs are highly flammable. For safe application, it is necessary to modify the flammability of GFRPs and reduce the smoke production and toxicity of gaseous products generated. These may be achieved with the addition of flame retardants and smoke suppressants [25]. 

Flame retardants are not a specific group of chemicals. They differ in structure and properties, but they all reduce the adverse impact of fires on people, the environment, and property. They can be used alone; however, to improve their effectiveness, the synergism effect between two or more flame retardants is often used. The addition of flame retardants helps fulfill the mandatory fire requirements for materials, but may also decrease their mechanical properties. The European product classification system, according to PN-EN 13501-1 in the field of reaction to fire (depending on its tendency for ‘burning’), is complex and expansive. Euroclasses have been introduced for floors A1fl, A2fl, Bfl, Cfl, Dfl, Efl, and Ffl and other construction products, namely A1, A2, B, C, D, E, and F. The product testing provides data, represented by the signs s1, s2, or s3, which indicate a tendency to release smoke, and can be translated as ‘a little or no smoke’ s1; ‘quite a lot of smoke’ s2; ‘substantial smoke release’ s3. Some construction products can melt and ignite to form flaming droplets. These ‘flaming droplets/particles’ tend to initiate new fires away from the original point of ignition, and must be considered when the products are used horizontally in ceiling or roof applications. The classification system ranks the level of release of flaming droplets/particles as d0 ‘none’; d1 ‘some’; and d2 ‘quite a lot’. Bringing all this information together indicates the complete Euroclass for a construction product as defined in BS EN 13501-1 Reaction to Fire.

**Figure 3:**
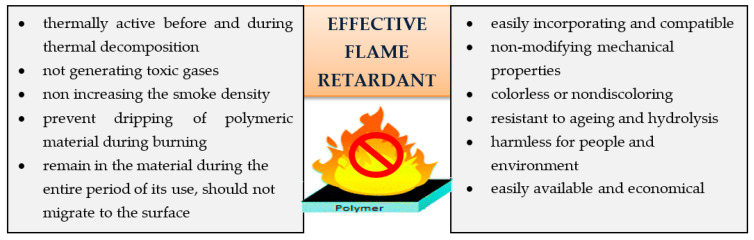
Quality properties of flame retardants [30].

In addition to that, there are also some more requirements for an effective flame retardant, as shown in Figure 3 [30].

Flame retardants contribute to the extension of time to ignition, slowing down the flame spread rating and generation of less toxic smoke during thermal degradation. They may act in the vapor and/or in the condensed phase. The two main types of flame retardants are additive and reactive. The former is mixed and dispersed in the matrix without bonding with polymer, whereas the latter is introduced into the polymer chain or is incorporated as a pendant group [31].

### 2.1. Halogenated Flame Retardants

Initially, UPR and glass/polyester laminates were modified with halogenated flame retardants. They were used as additive and reactive flame retardants [32]. The two main groups of halogenated flame retardants are chlorinated (CFR) and brominated (BFR). Their thermal stability increases from brominated aliphatic and chlorinated aliphatic to brominated aromatic [33]. Compounds such as tetrabromophthalic anhydride, tetrachlorophthalic anhydride, 1,4,5,6,7,7-hexachlorobicyclo[2.2.1]-hept-5-ene-2,3-dicarboxylic acid, or its anhydride were used [34]. Brominated bisphenols, polybrominated diphenyl ethers, hexabromocyclododecane [35], polychlorinated bisphenols, and alkanes have also been commonly used [36].

Only bromine- and chlorine-containing compounds have a commercial significance. The C–F bond in fluorine compounds is too strong and therefore these compounds do not interfere in the combustion process or are ineffective. Iodine compounds, although effective, are too loosely bonded to carbon. Both fluorine and iodine compounds are expensive.

BFRs can be divided based on their chemical structure, such as compounds with several benzene rings: polybrominated diphenyl ethers, decabromobiphenyl, 1,2-bis(pentabromophenyl); derivatives of tetrabromobisphenol acid: tetrabromophthalate diols and polyethers; derivatives of tetrabromobisphenol A (TBBPA): oligomeric and polymeric compounds TBBPA-carbonate oligomer, poly-di, and tribromostyrene. 

CFRs can be divided into aliphatic, cycloaliphatic, and aromatic compounds. The most commonly used aliphatic CFRs are chlorinated paraffins (CP) [37]. CP consist of chlorinated n-alkanes. Short-chains (SCCP) are C10-C13, medium-chains (MCCP) are C14-C17, long-chains (LCCP) are C > 17. The chlorine content is between 30% and 70% [38].

Another group of halogen-containing flame retardants are phosphorus flame retardants (PFRs). There are three main groups of PFRs: inorganic, organic, and halogen-containing. The first two are described in Section 2.3. The third group combines the properties of halogen and phosphorus atoms and includes compounds such as: tris(2-chloroethyl)phosphate (TCEP), tris(chloropropyl)phosphate (TClPP), tris(1,3-dichloro-2-propyl)phosphate (TDCPP), and tetrekis(2-chlorethyl)dichloroisopentyldiphosphate (V6). 

Halogenated FRs can be added and mixed with the polymer, or they can be chemically bonded to the polymer. They act in the gas phase and their effectiveness is dependent on the number of halogen atoms in a molecule. However, if the halogen and phosphorus atoms are present, they act independently [39].

The mode of action of these flame retardants is based on the inhibition of chain reactions occurring between the pyrolysis products and air. Hydrogen and hydroxyl radicals bind with the synthesized hydrogen halide, preventing reaction with oxygen and carbon monoxide, which results in the inhibition of the combustion [37]. They are very efficient in trapping free radicals. Other advantages are good miscibility, processability, and low cost [40].

However, UPRs modified with halogenated flame retardants usually generate dense, black smoke containing toxic and corrosive gaseous products such as hydrogen chloride, hydrogen bromide, or carbon monoxide. As a result, high smoke emissions may also cause evacuation problems due to a reduction in visibility [32]. Halogenated flame retardants pose a hazard to the environment and living organisms [40]. The discontinuation of the use of halogenated flame retardants is also due to environmental reasons. For example, the REACH regulation and the RoHS Directive have limited the usage of halogen-containing compounds [32].

#### Antimony Synergists for Halogenated Flame Retardants

The synergism effect occurs between certain types of flame retardants. It results in higher overall flame retardancy than the sum of effects obtained from single flame retardants. For example, it occurs, e.g., between resins containing halogen atoms or halogenated flame retardants and antimony trioxide Sb_2_O_3_. This combination reduces flammability, but the partial replacement of an antimony trioxide to zinc borate, for example, significantly reduces the intensity of smoke emission [41]. The combustion of halogen-containing compounds with antimony trioxide is shown in the following reactions [42]:

$$R-X+P-H\;\to H-X+R-P\;Sb_{2}O_{3}+2\;HX\to 2\;SbOX+H_{2}O\;5\;SbOX\to Sb_{4}O_{5}X_{2}+SbX_{3}\;4\;Sb_{4}O_{5}X_{2}\to 5\;Sb_{3}O_{4}X+SbX_{3}\;3\;Sb_{3}O_{4}X\to 4\;Sb_{2}O_{3}+SbX_{3}$$

Antimony trioxides hinder reactions in the gas phase by activating through a progressive release of halogenated radicals [43]. Although antimony trioxide suppresses the flammability and reduces carbon monoxide emission, it is not commonly used for UPRs [44]. This is because, among other reasons, both halogenated flame retardants, as well as their antimony synergists, have an adverse effect on the environment [43]. 

### 2.2. Inorganic Hydroxide Flame Retardants

Other flame retardants which meet the requirements of regulations are inorganic hydroxides. Aluminum trihydroxide (ATH) Al(OH)_3_ and magnesium hydroxide (MH) Mg(OH)_2_ are the main inorganic fillers. Their mode of action is based on releasing water molecules under thermal degradation, which is called endothermic dehydration. This results in heat absorption and a reduction in temperature. Released water molecules dilute the concentration of flammable gases and reduce the amount of free radicals. Synthesized metal oxides form a heat barrier layer on the surface of the material [45]. 

The type of hydroxide used is contingent on the processing temperature of the material. Degradation of ATH occurs at 190–230 °C, whereas, for MH, it is ca. 320 °C. Hence, the latter is used in UPRs that show higher thermal stability. 

Hydroxides’ advantages are nontoxicity, a reduction in polymer temperature because of heat absorption, and the reduction in the acidity of combustion products [46]. MH is also a smoke suppressant [47]. However, the major disadvantage is the necessity of adding them in a large amount. These flame retardants are highly efficient when their content in the material is higher than several dozen percent. Tang et al. showed that UPR modified with 55% of MH is more flame-resistant and has better smoke suppression properties than composites with 35% and 45% of additive [48]. However, this may negatively impact the material’s mechanical properties and may cause problems during processing [32]. The research shows that the addition of ATH in an amount that causes a reduction in heat emission is sometimes too large and prevents the fabrication of some elements [49]. When inorganic hydroxides are used, the density of the material increases and so they need to be reduced by adding a hollow filler [50]. Limitations regard not only the processing but also the glass fiber content. When this exceeds 30%, the large amount of flame retardant may lead to the further deterioration of mechanical properties [51].

ATH and MH may be used in a mixture. The research shows that composites with UPR and both hydroxides have improved thermal stability and reduced mass loss rate during decomposition. The best results are obtained when the mass ratio of ATH and MH is 4:1 [47].

To significantly reduce the heat release rate, the synergism effect between ATH and some phosphorus-containing flame retardants may be used. It is shown that crystalline ATH shows greater synergistic activity than amorphous ATH when used with aluminum hypophosphite or zinc diethylphosphinate [52]. Hörold and Arnsmann have shown that the use of nitrogen compounds also increases the efficiency of inorganic hydroxides at lower contents [53].

### 2.3. Phosphorus Flame Retardants

**Figure 4:**
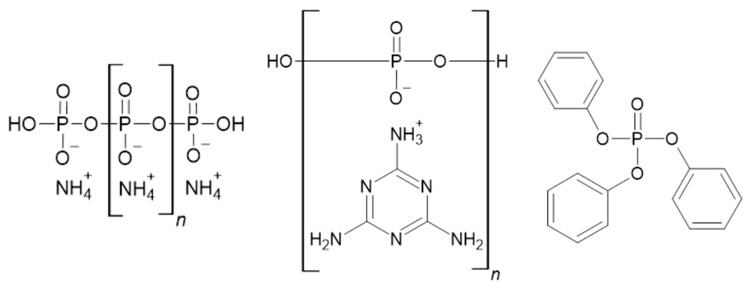
Structures of ammonium polyphosphate [58], melamine polyphosphate [59], and triphenyl phosphate [60].

Red phosphorus, inorganic phosphates, and numerous organophosphorus compounds are often used flame retardants [54]. There is a growing interest in triphenyl phosphate (Figure 4) because of its high efficiency in producing a protective charred layer [55]. An example of an additive phosphorus flame retardant is tris(allyloxymethyl) phosphine oxide (TAOPO) containing a P–C bond. Lin et al. showed that TAOPO, when used as a co-curing agent, significantly increases thermal stability and reduces the peak heat release rate and total heat released [56]. Also worth mentioning is ammonium dihydrogen phosphate, which is not added into the polymer matrix but is useful as a glass fiber coating for GFRP composites. However, when flammability properties significantly improve at contents of about 20wt%, the interfacial adhesion and wetting decrease somewhat [57]. Figure 4 shows structures of flame retardants from that group.

Phosphorus-containing compounds can interact not only in the solid phase affecting the thermal decomposition of material and the charring process. They can also interact in the gas phase causing the capture of free radicals [61]. Reduction–oxidation reactions of hydrocarbons are slowed down or interrupted and, therefore, the amount of heat released is lower. The efficiency of phosphorus flame retardants in a gas phase is comparable to halogenated flame retardants [62]. 

The mode of action varies for different groups of phosphorus flame retardants. For example, aluminum diethyl phosphinate shows higher activity in the gas phase, releasing diethylphosphinic acid, than in the condensed phase. Only a small amount of this flame retardant takes part in residue formation. Phosphate-based flame retardants act as acid precursors, leading to esterification and dehydration processes, resulting in char formation. However, when phosphate esters are released into the gas phase in place of reacting with the polymer, they also show a significant flame-inhibiting effect. Thus, phosphate-based phosphorus flame retardants, as well as red phosphorus, can often act in the condensed phase taking part in the char formation and in the gas phase-inhibiting flames [63]. However, it is worth pointing out that flame inhibition results in a less complete combustion, which is a reason for increasing CO and smoke production. 

There are many possible reactions taking place in the gas phase, where hydrogen and hydroxy radicals are being replaced or rendered harmless. PO-radical is pointed out as the one playing the main role in the gas phase. The most important examples of reactions are shown below [62].

$$PO•+H•\;\to HPO\;PO•+OH•\;\to HPO_{2}\;HPO+H•\;\to H_{2}+PO•\;OH•+H_{2}+PO•\;\to H_{2}O+HPO\;HPO_{2}•+H•\;\to H_{2}O+PO\;HPO_{2}•+H•\;\to H_{2}+PO_{2}$$

$HPO_{2}•+OH•\;\to H_{2}O+PO_{2}$ the condensed phase dehydration of the polymeric structure occurs. After that, reactions such as cyclization and aromatization take place, as well as cross-linking initiated by phosphorus compounds or their decomposition products. Inorganic phosphate glass is formed and probably also phosphoric acid [56].

It is shown that molecule structure, the process of decomposition, and interaction with the polymeric matrix strongly influence the effectiveness of these flame retardants [63]. In contrast to inorganic hydroxides, they are effective at lower contents in the material [64]. In addition to the flame retardancy effect, they may cause a reduction in smoke emission [65].

Phosphorus-containing flame retardants have some disadvantages. Red phosphorus, when nonencapsulated, may react with moisture and form highly toxic phosphine. This may occur during the melting process because of its poor thermal stability. Organophosphorus flame retardants may undergo volatilization and migration to the surface during the processing [66].

### 2.4. Nitrogen Flame Retardants

Nitrogen compounds such as melamine and its derivatives, triazines, isocyanates, urea, guanidine, and cyanuric acid derivatives without phosphorus atoms in molecules are also used as flame retardants for certain polymers [54,67,68]. They are environmentally friendly (they are less toxic) and, importantly, materials containing these compounds are suitable for recycling [69]. However, melamine may cause serious hazard to people’s health [70]. 

**Figure 5:**
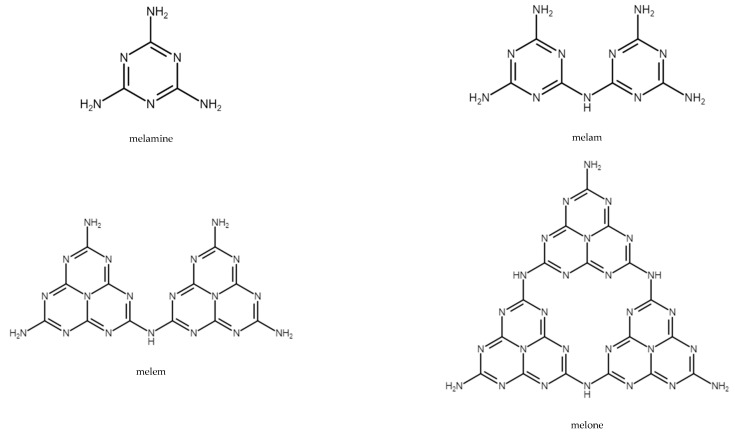
Structures of melamine, melam, melem, and melone [73].

Melamine is nitrogen-rich and contains 67% of nitrogen by mass. It sublimates at 350 °C, absorbing large amounts of heat, resulting in a decrease in temperature. Melamine decomposes with the release of ammonia, which reduces the concentration of flammable gases and leads to the formation of thermally stable condensates such as melam, melem, and melon. Figure 5 shows the structures of melamine and its condensates. 

Melamine cyanurate, an adduct of melamine and cyanuric acid, undergoes thermal dissociation into its components, and then forms melamine condensates, as if used alone. The oxidation reaction competes with condensation, which is more favored when melamine salts are used [71,72]. Most salt-structures of melamine and related substances are active in the condensed phase through endothermic decomposition, the dilution of combustible gases, and the formation of the char layer [72].

To meet adequate effectiveness in fire retardancy, the content of melamines must be very high, even up to 65% *w*/*w*, which reduces mechanical properties of the end products. It may also cause difficulties in processing [74].

### 2.5. Phosphorus–Nitrogen Flame Retardants

One of the most efficient groups of flame retardants for UPRs concerns phosphorus- and nitrogen-containing compounds. Ammonium polyphosphate (APP) and melamine polyphosphate (MPP) (Figure 4) are frequently used because of the synergistic effect occurring between phosphorus and nitrogen. These flame retardants undergo thermal decomposition to phosphoric acid.

$${\left(NH_{4}PO_{3}\right)}_{n}\overset{>250\;{}^{\circ}C}{\to }{\left(HPO_{3}\right)}_{n\;}+nNH_{3}\;\mathrm{polyphosphoric}\;\mathrm{acid}\;{\left(HPO_{3}\right)}_{n}+polymer\overset{-H_{2}O}{\to }\left(HPO_{4}\right)+char$$

Then, polyphosphoric acid is formed, which undergoes esterification and produces a protective layer [75]. Mixing phosphorus- and nitrogen-containing compounds, APP and MPP, with nanofillers enhances the flame retardancy of UPRs [76]. Research shows that the optimum content of compounds such as APP in composites based on UPR is ca. 30 parts per hundred rubber (phr) [77].

A bio-based phosphorus and nitrogen flame retardant for UPR was investigated by Farishi et al. Hydroxyl groups of lignin were converted to phosphate nitrogen during reactions. The addition of 12.5% of modified lignin resulted in a significant increase in thermal stability compared to neat lignin. However, it was shown that the amount of char residue did not differ that much [78].

### 2.6. Boron Flame Retardants

Boron flame retardants are used alone as well as with other flame retardants. They may be used instead of antimony flame retardants or mineral fillers. Boron compounds show synergistic or adjuvant effect with, e.g., mineral fillers such as ATH and MH, phosphorus–nitrogen flame retardants as ammonium phosphate, and APP-based intumescent systems.

Actions in both condensed and gas phases make boron compounds widely used in various polymeric materials. Enhanced char formation and its stabilization, suppression of dripping, and improvement of the barrier effect due to sintering is observed in the condensed phase. In addition, inorganic boron compounds undergo endothermic decomposition, releasing water, which results in fuel dilution. Worth mentioning are some organoboron compounds that liberate free radicals and noncombustible gases, rather than water [79]. 

Boron compounds are also fine smoke suppressants. This is due to an occurring char-forming catalytic effect at relatively low temperatures, forming glossy films. In addition, released products contain less toxic gases, especially CO [80].

As demonstrated in the literature, highly efficient flame retardants for chlorinated polyester resins are melamine borate (C_3_H_6_N_6_) · 2B(OH)_3_ or ammonium pentaborate NH_4_B_5_O_8_ · 4H_2_O. The positive effect of reducing flammability comes from producing boric acid H_3_PO_3_ and forming a protective layer [81]. Boron compounds such as zinc or calcium borates also show a synergism effect with halogenated flame retardants [82]. Studies of the addition of compounds such as zinc borate, boric acid, or boron trioxide to resins containing red phosphorus show a reduction in the heat release rate and in the total heat released [83]. However, Belausova et al. have showed that some boron compounds can be effective flame retardants without other additives such as halogenated flame retardants or antimony oxides [84].

A recent trend in the flame retardancy market concerns the use of nanosheets. Chu et al. used two-dimensional boron nitride sheets to improve the fire safety and impact strength of UPR composites. The increase in the thermal stability and Limiting Oxygen Index (LOI) value were shown, as well as a decrease in the peak heat release rate and total heat release [85]. Wang et al. used phosphorus, nitrogen, and silicon-co-contained boron nitride nanosheets, and this also resulted in a significant decrease in peak heat release rate and total heat release. Additionally, the production of toxic volatiles including aromatic and carbonyl compounds and carbon monoxide was highly decreased [86].

The advantage of using boron flame retardants is that their low content in the material (about 10%) does not affect the mechanical properties [81]. In addition, boron compounds are economically beneficial [87]. Some papers have suggested that they are also environmentally friendly, of low mammalian toxicity, of low volatility [88], and less toxic than other flame retardants [87]. However, in high concentrations, boron flame retardants can be toxic to aquatic and terrestrial organisms [89]. 

### 2.7. Tin and Zinc Flame Retardants

Initially, tin–zinc compounds were applied to resins containing halogen atoms due to the synergism effect occurring between them. Thereafter, it was shown that compounds such as tin zinc oxide (ZS) ZnSnO_3_ or tin zinc hydroxide (ZHS) ZnSn(OH)_6_ are also effective flame retardants when applied to halogen-free UPRs [90]. The mode of action of tin is based on capturing free radicals, as shown in the following reactions:

$$SnO_{2}+H_{2}\to SnO+H_{2}O\;SnO+H^{*}\to SnOH^{*}\;SnOH^{*}+H^{*}\to SnO+H_{2}$$

In addition, tin acts as a char promoter and is a catalyst for flame soot oxidation processes. Despite this, compounds containing only tin atoms such as tin oxide SnO_2_ are less effective flame retardants than tin–zinc compounds [91]. On the other hand, the addition of zinc oxide ZnO to UPR reduces the activation energy required to start the degradation of material [92]. Between ZnO and ATH, there is a synergism effect. Ugal and Jimaa showed that the addition of a mixture of these flame retardants to the UPR results in an increase in the LOI and a decrease in both the maximum flame height and rate of burning [93]. Nageswara Rao et al. reported that ZnO used with intumescent flame retardants also improved LOI, even when its content is only 3% *w*/*w*—it increases thermal stability because it promotes charring at high temperatures [94].

ZHS and ZS form a coating that prevents heat transport into the material. In addition to reducing flammability, these compounds also reduce smoke [46] and carbon monoxide emissions and sustain the charring process [95]. Kandare et al. investigated the thermal behavior of APP, ZS, ZB, ZHS, and nanoclay composites with UPR. They show that the mixture of tin–zinc compounds and APP improves thermal stability and promotes charring. The addition of nanoclay to that mixture can further improve thermal stability. Due to the endothermic decomposition of nanoclay, the temperature decreases, preventing depolymerization reactions [96]. 

However, problems in polymer processing may occur, especially in obtaining a homogeneous mixture of polymer matrix and tin–zinc compounds, as they tend to accumulate [97]. Due to their high price, they are often used as coatings for cheaper inorganic mineral fillers. This reduces costs and reduces the amount of additive used [98].

Other noteworthy zinc-containing flame retardants are its compounds with boron. There is a wide range of binary zinc borates of general composition; a ZnO · b B_2_O_3_ · c H_2_O. 2 ZnO · 3 B_2_O_3_ · 3 H_2_O or Zn[B_3_O_4_(OH)_3_] is used in most applications. In addition, biocidal registrations are carried by certain brands for this compound only [41]. 

The mode of action of zinc borate (ZB) is to change the pyrolysis process. ZB releases water at high temperatures and forms boric acid, which enhances the compact structure of char [99]. Zinc borate is often used with ATH and MH in halogen-free systems because of the synergism effect. At 600 °C, a ceramic-like layer is formed which protects the unburnt polymer. In addition, water released at high temperatures of 290–450 °C contributes to forming intumescent char and inhibits flame combustion. The mode of action of ZB with ATH and MH is summarized by following reactions [100]:

2 ZnO · 3 B_2_O_3_ · 3.5 H_2_O + Al(OH)_3_ → x Al_2_O_3_ · y ZnO · z B_2_O_3_ + B_2_O_3_ + H_2_O↑

2 ZnO · 3 B_2_O_3_ · 3.5 H_2_O + Mg(OH)_2_ → x MgO · y ZnO · z B_2_O_3_ + B_2_O_3_ + H_2_O↑

ZB, when used with halogenated systems, formed zinc species that catalyze dehydrohalogenation and enhance the crosslinking process. This causes an increase in the thickness of the char layer formed, a reduction in smoke emission intensity, and the suppression of the smoldering. In addition, the boron trioxide released during decomposition stabilizes the char layer and inhibits afterglow [100,101]. The mode of action is summarized by following reaction [100]:

2 ZnO · 3 B_2_O_3_ · 3.5 H_2_O + HCl → ZnCl_2_ + Zn(OH)Cl + B_2_O_3_ + H_2_O↑ + BCl_3_

### 2.8. Nanoclays as Flame Retardants

Nowadays, nanoclays are receiving much attention as flame retardant additives. They are a class of inorganic clay-based nanomaterials able to integrate with various materials. Montmorillonite (MMT), bentonite, kaolinite, hectorite, and halloysite form silicate or aluminum–silicate structures [102].

Unlike conventional flame retardants, nanoclays are harmless to the environment, highly effective, and, above all, the addition of nanoclays can impart specific properties to the material [103]. Studies conducted on resins modified with nanoclays such as MMT have shown not only a reduction in flammability but also an improvement in strength properties [104]. Smaller particle sizes also reduce the mobility of carbon chains, which affects the volume, surface, and properties of the resulting nanocomposite [105]. 

MMT is one of the most widely used nanoclays for UPRs. Its surface area and ratio of the longest and shortest size are very high. This results in enhancing flame retardancy, but it occurs only when a good dispersion is achieved. Otherwise, a reduction in the specific surface area may cause a decline in flame retardant properties.

**Figure 6:**
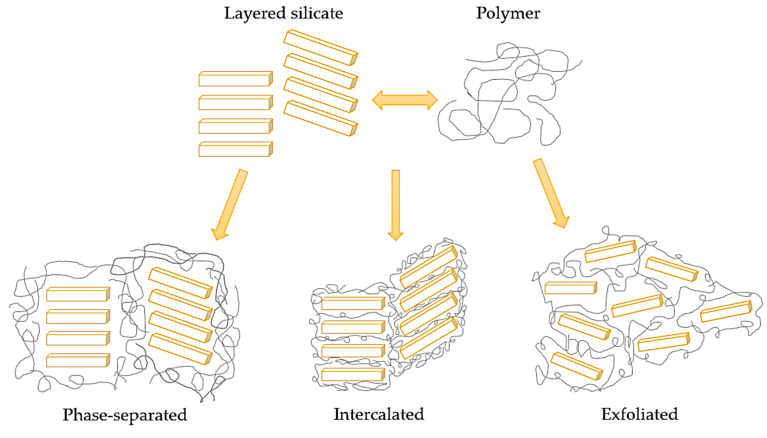
A scheme of dispersion states of the clay in the polymer matrix [106].

There are three dispersion states of the clay: phase-separated, intercalated, and exfoliated. The last one is favored due to its higher specific surface area. All of the states are shown in Figure 6 [106]. 

The large specific surface area of nanoclays allows for a reduction in the amount of additive even to a few percent. The mode of action is based on char layer formation that blocks heat transport. For inorganic nanoclays, capturing free radicals is common [25]. Nanocomposites containing silicates or silica exposed to high temperatures remain intact and the decomposition of organic parts takes place inside the material, thus reducing the amount of soot released [61]. The insulation of the material and retardation of the mass loss occurs due to the build-up of a carbonaceous–silicate char layer. It is generally observed that nano flame retardants reduce the flammability by enhancing char formation and minimizing the heat release rate [107].

**Figure 7:**
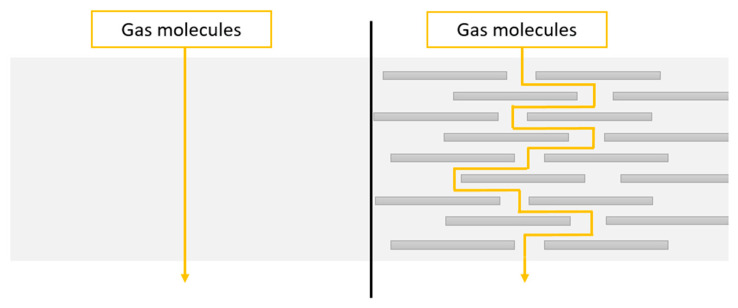
A scheme of a tortuous pathway in a polymer matrix without (left side) and with exfoliated nanoplatelets (right side) [109].

If silicate layers of nanoclays are suitably intercalated or exfoliated in the matrix, the physical formation mechanism is more efficient. The tortuous path formed by these layers restricts the diffusion of volatile species released during the combustion. This is called a labyrinth effect [108]. A scheme of a tortuous pathway is shown in the Figure 7.

There is a synergism effect between nanoclays and some conventional flame retardants. This has been observed for extensible graphite, synthetic nanosilica, and phosphorus–nitrogen compounds. Simultaneous application of both retardants results in the faster formation of the protective char layer inhibiting the flow of oxygen and reducing the emission of low molecular weight compounds formed during the decomposition of plastic [110].

### 2.9. Intumescent Flame Retardants

One of the recent methods for reducing UPR flammability is the addition of compounds that form intumescent char under the influence of heat. The char residue forms a thermal barrier and reduces the transport of heat into the material [46]. This phenomenon occurs during the material’s thermal decomposition in the solid phase and results in the release of gaseous products. The surface of the plastic is being simultaneously charred and foamed [111]. The foam produced initially has an organic–inorganic structure, however the fire changes it into an inorganic one [112]. 

In order to form a porous layer on the surface of the material, the intumescent system has to contain at least three types of compounds: a carbonizing source, an acid source, and a blowing source. The carbonizing source can be polyhydric alcohols (pentaerythritol), carbohydrates (sorbitol, mannitol), poly(carbohydrates) (starch), phenol–formaldehyde resins or char-forming polymers. Phosphoric acid, boric acid, sulphuric acid, and halides, as well as their derivatives, are used as an acid source. Mostly nitrogen-containing compounds such as melamine, urea or dicyanamide, that decompose with the release of large amounts of non-flammable gases such as nitrogen, carbon dioxide, or ammonia are used as blowing agents. 

Decomposition of an acid source results in the formation of a strong inorganic acid. It promotes the dehydration of a carbonizing agent in production of the carbonaceous layer [113,114]. At the same time, the degradation of blowing agent occurs. Released inflammable gases expand the carbonaceous layer and provide the formation of a swollen multicellular layer, protecting from heat, flames, and oxygen [114]. Table 2 presents examples of intumescent paint compositions (1–3) and intumescent UPR composites (4). 

The efficiency of char formation is related to polymer structure. Degradation of the polymer itself is also a source of carbon, double bonds, and aromatic rings, which leads to increased char production during combustion. The mentioned inorganic oxoacids accelerate the char formation process, influence the flame propagation process, and may show other important effects such as heat sink or smoke retarding [119]. In the first stage, acid-catalyzed depolymerization occurs, whereas, in the second, there is dehydration. As a result, double bonds are formed at the end of polymer chains, which influence the formation of an intumescent char layer. The course of the reactions is presented below [120].

$$\cdots CH-CH_{2}-OH+H^{+}\to \cdots CH-CH_{2}-OH_{2}^{+}$$

$$\cdots CH-CH_{2}-OH_{2}^{+}\to \cdots C=CH_{2}+H_{2}O+H^{+}$$

$$\cdots CH-CH_{2}-OH+H_{3}PO_{4}\to \cdots CH-CH_{2}-O-P{\left(OH\right)}_{2}O+H_{2}O$$

$$\cdots CH-CH_{2}-O-P{\left(OH\right)}_{2}O\to H_{3}PO_{4}+\cdots C=CH_{2}$$

Such a modification of plastics with an intumescent flame-retardant system results in a significant reduction in both heat release during the thermal decomposition of the polymer and flammable gas emission [121]. However, the disadvantage of these systems is the possibility of their migration to the polymer surface due to low mass [122].

Intumescent flame retardants, when used with clay minerals such as montmorillonite or kaolinite, show the synergism effect and are highly effective in improving fire resistance. When UPR is modified with an intumescent system, a porous carbonaceous foam is formed. With the addition of modified montmorillonite on the surface of this foam, a ceramic-like structure is formed, which causes the char layer to be denser and more compact [122].

The surface protection of the composite may also be achieved by the use of an intumescent mat, which acts as a thermal barrier, providing composite structural integrity. As distinct from additive flame retardants, fabrics or coatings do not affect the crosslinking process, and, therefore, do not change the mechanical properties of the composite. Usually, the composition of mats is similar to intumescent systems and contains an acid source, an inorganic acid, a carbon source, and a blowing agent. Research shows that these mats improve the fire resistance of most GFRP composites: the peak heat release rate, the fire growth rate, and the rate of temperature increase inside the composite are lower when mats were used. However, some difficulties may occur in reducing time of ignition in order to achieve better fire protection at later fire stages [123]. 

Another commonly used material in passive fire protection is expandable graphite, which may be intercalated with inorganic or organic acids. This thermally decomposes, producing a large quantity of gases. The perpendicular direction of gases released to the exposed surface allows for the formation of a protective char layer, which not only inhibits the diffusion of oxygen and heat into the material, but also reduces the heat emitted from the material by trapping combustible volatiles. Insulative surface barriers can be applied with other flame retardants. The improvement in some flammability properties occurs probably due to a synergism effect between flame retardant decomposition products and intumescent mat and/or physical processes during decomposition. However, the total heat released is not reduced for that matter [124].

The latest research focuses on titanium compounds. Complex solid super acid-containing titanium may be a substitute for the usual acid source in intumescent flame retardant systems. This improves the flame retardance of composites by reducing the heat release rate, total heat release, smoke production rate, and the emission of CO and CO_2_. The limited oxygen index of these composites is significantly higher, and thermal stability is increased [125]. Polymetallic core-shell nanospheres containing titanium also improve some flammability properties. Heat release parameters are only slightly improved, but smoke parameters such as total smoke production and the peak smoke production rate are significantly reduced. Composites prepared with core-shell architectures release fewer toxic products during thermal decomposition: specifically, the total CO release is reduced. In addition, some mechanical properties such as tensile and impact strength are improved [126].

### 2.10. Polyhedral Oligomeric Silsesquioxanes

**Figure 8:**
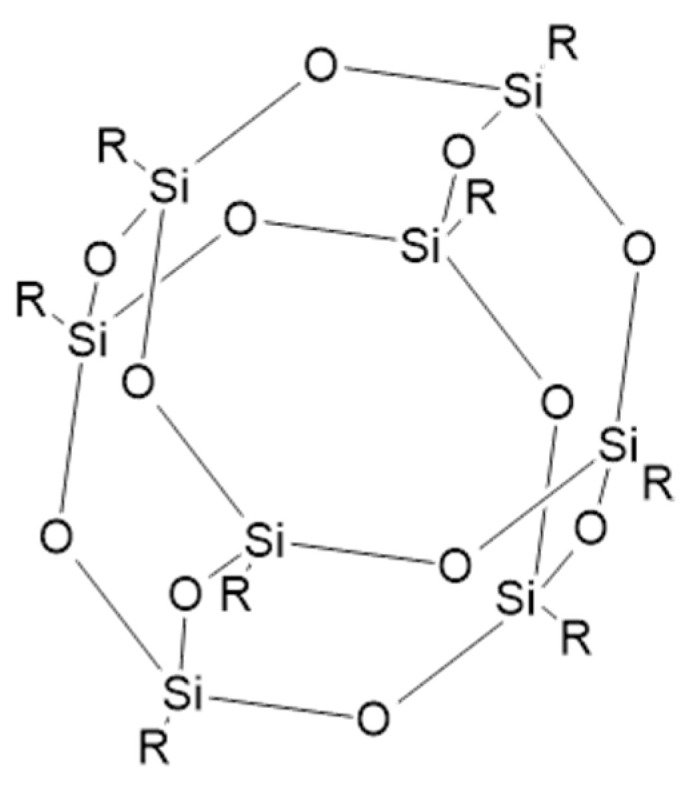
A structure of polyhedral oligomeric silsesquioxanes (POSS) [128].

Recent trends in the flame retardant market focus on three-dimensional nanostructure material such as polyhedral oligomeric silsesquioxanes (POSS). Their general formula is (RSiO_1,5_)_n_, where R is the alkyl, aryl, or hydrogen group [127]. The cage-like structure of POSS is presented in Figure 8.

The inorganic backbone accounts for thermal stability and inflexibility, whereas substituents are important in terms of the interaction with the polymer, as they allow POSS to dissolve in polymers, solvents, or coatings. These functional groups may be reactive (alkylene, hydroxy) or nonreactive (hydrogen, alkyl). The former can homo and copolymerize with other structures, and the latter groups make POSS compliant with polymers. Thus, POSS derivatives can be bonded or physically blended with polymers [129]. In regard to flame retardancy, two main types of POSS have been studied. For the first one, if containing all the same substituents, then the R group can be methyl, phenyl, isobutyl, or isooctyl; the second one contains the same seven aforementioned substituents and one functional group that can be ester, silane, isocyanate, methacrylate, alcohol, epoxide, or amine. Functional groups improve the compatibility between nanocages and the matrix, as well as enable the occurrence of chemical grafting or polymerization reactions. Due to the variety of these substituents, POSS can be modified according to the polymer matrix’s requirements [71]. 

POSS, when modified with functional groups, may influence fire retardant properties in two ways: The first way is by undergoing oxidative degradation initiated by heat, which has an impact on the structure and morphology of the char layer. The other way is by conducting chemical and physical side reactions, mitigating the combustion [130].

The POSS loading at which a significant improvement of flammability is observed is less than 10 wt%. Despite low modifier loadings, and its tendency to aggregate, it is possible to achieve satisfying dispersion when POSS substituents are compatible with the polymer matrix [131]. POSS can form strong bonds with the surface of the fillers and break particle–particle interactions in highly-filled resins. This results in a reduction in the viscosity and the enhancement of mechanical properties by means of increasing the strength, modulus, and rigidity [132]. The POSS mode of action is based on forming a protective thermal barrier. Firstly, after ignition, POSS migrate to the surface of the material. The initial decomposition provides for the creation of a ceramic char layer consisting of Si–O bonds fused together, insulating the material from heat and gas access. It can also reduce smoke production and the release of toxic gaseous products. Slightly different mechanisms may occur when different side groups are used [131].

Glodek et al. investigated the fire resistance of vinyl ester resins modified with methacrylisobutyl POSS and methacryl POSS. The research showed that the second one slightly increased fire retardancy. However, due to the high cost of the additive and the too-low efficiency, it was not taken into consideration [133]. Polyester-based nanocomposites prepared via in situ polymerization containing POSS-modified graphene oxide were also investigated. The thermal decomposition temperature was increased by nearly 70 °C at 10% mass loss. Additionally, some mechanical properties such as tensile strength and electrical conductivity were significantly improved [127]. An increase in the thermal decomposition temperature of 35 °C was observed for composites modified with 5 wt% of POSS-MMT. Overall, the ductility of the polymer and tensile strength was improved, but a slightly better improvement was observed for composites containing 1 and 3 wt% of the modifier due to the agglomeration of nanofillers at higher loads. The enhancement of electrical properties related to POSS-MMT acting as a conducting network was also noticed [134]. 

## 3. Environmental Impact of Flame Retardants

Flame retardants are anthropogenic pollutants. They are plastic additives used in many different fields of industry, including electronics, textiles, and automotive [135]. 

The golden age of flame retardants was between 1950 and 1980, when fire safety regulations were established in Europe and the United States. During this period, effective flame retardants meeting the requirements were searched for. Initially, their potential toxicity and negative impact on the environment were not considered. This changed when articles on the carcinogenic and mutagenic properties of brominated flame retardants began to appear in various journals [135,136].

**Figure 9:**
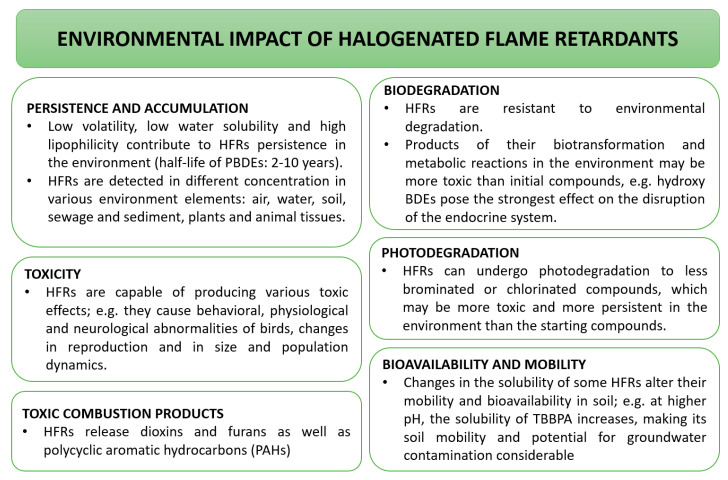
Environmental impact of halogenated flame retardants [135,138,139,140].

The most economical are halogenated flame retardants. These include compounds containing chlorine or bromine: tetrabromobisphenol A (TBBPA), hexabromocyclododecane (HBCD), and polybrominated diphenyl ethers (PBDEs). These compounds may pollute air, water, and soil. They are present in river sediments and muds, as well as in bivalves, fish, crustaceans, amphibians, reptiles, birds, mammals, and human tissues [137]. Therefore they are persistent and not fully biodegradable, and thus accumulate in the environment. In addition, some of them may only be partially biodegradable, resulting in the formation of compounds that are often more damaging [135]. The environmental impact of halogenated flame retardants is described in Figure 9.

SCCP, because of their environmental persistence, bioaccumulation potential, and toxicity, have been registered in Stockholm Convention Persistent Organic Pollutants (POPs) since 2017. Despite the fact that medium- and long-chain homologue groups may also have an adverse effect on the environment, they were used as a replacement for the banned substances. All the CPs may migrate from other materials, resulting in the contamination of environmental matrices and indoor dust. When used in sporting equipment and toys, exposure via direct dermal contact had to be considered. SCCPs exhibit neurotoxic and endocrine disrupting properties, and they are also classified as potential carcinogens. MCCPs’ toxicity is similar [38].

According to the Registration, Evaluation, Authorization and Restriction of Chemicals (REACH), dibromostyrene regulation is suspected of being hazardous to the aquatic environment and being persistent in the environment. 3-bromostyrene is also suspected of being carcinogen, hazardous to the aquatic environment, in addition to being a respiratory sensitizer and a skin irritant [141].

Polybrominated diphenyl ethers are a family of chemicals which have been used as flame retardants. A number of commercial products containing POP-PBDEs have been placed on the market in the Union (such as DE-71, Bromkal, Saytex137), which are categorized based on their major homologue groups called “Commercial Pentabromo diphenyl ether (C-PentaBDE)”, “Commercial Octabromo diphenyl ether (c-octaBDE)’, and ‘Commercial Decabromo diphenyl ether (C-decaBDE)’. A growing concern for the environmental and health effects of PBDEs, based on a weight of evidence, particularly for lower order homologue groups, led to a ban of the C-PentaBDE and c-octaBDE in the Union in 2004 (by Directive 2003/11/EC). Since June 2009, these restrictions for c-pentaBDE and c-octaBDE have been included in REACH Annex XVII on the restrictions on manufacturing, placing on the market, and the use of certain dangerous substances, preparations, and articles by the Regulation 552/2009. Furthermore, in 2009, the chemical homologues tetra, penta, hexa, and heptaBDE were added to Annex A (banned) of the Stockholm Convention at the fourth conference of the parties (COP-4). While the c-pentaBDE and c-octaBDE were banned in 2004, the use of c-decaBDE was permitted to continue. For C-decaBDE, this included an agreement that c-decaBDE should not contain less than 97% *w*/*w* of the decaBDE homologue. Subsequently, a growing weight of evidence has continued to develop around the environmental and health effects of decaBDE, particularly in its capacity to degrade within the environment to form lower order homologue groups through a process of debromination. Following a nomination by Norway (in 2013), decaBDE was added to the Stockholm Convention under Annex A (banned) at the COP-8 in 2017. In the Union, decaBDE was added to the candidate list for substances of very high concern (SVHC) under the REACH Regulation in December 2012. In 2014, the Norwegian Environment Agency submitted a proposal for decaBDE to be added to Annex XVII of REACH (Restriction). In February 2017, decaBDE was added to Annex XVII of REACH (by Commission Regulation (EU) 2017/227). This restriction prohibited the substance decaBDE from being used at greater than 0.1% *w*/*w* in the manufacturing process, or when placing it on the market in another substance as a constituent, a mixture, or an article or any part thereof after 2 March 2019. Finally, decaBDE was listed in the POPs’ Regulation in 2019, which further restricted its use in the Union in line with the Stockholm Convention and replaced the restriction in Annex XVII of REACH. Phosphorus flame retardants were supposed to be a good substitute for the phased out BFRs from the market. However, research shows that they are now detected in various elements of the environment (e.g., air, water, dust, sediment, biota, and food). According to the research presented by Pantelaki and Vouts [142], the presence of PFRs has been confirmed in marine and river waters (and sediments), sewage and sewage sludge, and even in drinking water. The most frequent and abundant compounds from the halogenated PFRs were TClPP and TCEP. The presence of these compounds in surface waters may be caused by atmospheric transport, and their presence in wastewater may be caused by their wide application, as well as by their stability during biological treatment processes. 

In animal studies, it was found that Cl-PFRs can accumulate in the liver and kidneys (e.g., TCPP), disrupt the functions of the endocrine system and reproductive functions (reduced fertility), and increase developmental defects (e.g., TDCPP) [143], disrupting the development of the nervous system (e.g., TClPP); many of them are carcinogenic (especially Cl-PFRs, except V6) [39,143]. TCEP, TCPP, and TDCPP are believed to exhibit neurotoxicity (associated with motor deficits and dopaminergic degeneration), cytotoxicity (reduced viability and morphological changes in human peripheral blood mononuclear cells), and developmental toxicity (associated with growth inhibition in zebrafish offspring [144,145] in relation to animal organisms.

The increasing amount of data on the impact of HPFRs on human health shows that they can accumulate in the liver and kidneys, and such compounds as TCIPP and TDCIPP lower the level of human hormones and affect reproductive capacity (reduction in sperm quality) [146] and are potentially carcinogenic. 

Luo et al. [144,145] proved that exposure of women in the third trimester of pregnancy to bis (1,3-dichloro-2-propyl) phosphate (BDCIPP) may result in the impaired growth of the fetus, which is at risk of developmental toxicity (malformations).

Antimony trioxide, used frequently as a synergist for halogenated flame retardants, is a substance of concern. The health risks of Sb_2_O_3_ include both short- and long-term effects. In addition, it is possibly a human carcinogen [43]. According to Classification, Labelling and Packaging (CLP) regulation, antimony oxide causes health hazards, and, for the environment, it is toxic to aquatic life, with long-lasting effects [147]. In addition, diantimony trioxide is suspected of causing cancer [148]. The BlueSign^®^ label also limited the use of antimony, its salts, and compounds. Criteria for the chemical assessment of flame retardants are high and include environmental, health, and safety aspects [149].

Inorganic flame retardants such as aluminum hydroxide and magnesium hydroxide pose no threat to the environment. They do not contaminate it and are nontoxic [150].

Inorganic and organic phosphorus flame retardants are widely and commercially used. The least problematic phosphorus flame retardants are red phosphorus and ammonium polyphosphate [39]. The organophosphate flame retardants (OPFRs) are used as a replacement for polybrominated diphenyl ethers, which carry a high environmental burden [151]. They are not bounded with the polymer, thus they easily release into the environment by volatilization, abrasion, and dissolution. Recent studies show their presence in aquatic, terrestrial, and atmospheric environments. The Danish Environmental Protection Agency has voiced concerns about the presence of OPFRs, in particular the chlorinated phosphate ester cluster in children’s toys; 5 mg kg^−1^ is a limit value for their presence [142]. The Environmental Protection Agency designated triphenyl phosphate (TPP) as a high-priority substance in 2019. At present, it is undergoing risk evaluation for causing hazards to aquatic and terrestrial organisms [152]. According to REACH, it is very toxic to aquatic life, with long-lasting effects. It is also under assessment because of its endocrine-disrupting capacity [153]. Trixylyl phosphate also is registrated by the European Chemicals Agency, as it may damage fertility or unborn children. In addition, it is also very toxic to aquatic life and causes long-lasting effects. It can cause damage to organs through prolonged or repeated exposure. It is under evaluation for being toxic to reproduction and as well as being persistent, bioaccumulative, and toxic [154]. The BlueSign^®^ label banned several phosphorus flame retardants, e.g., bis(2,3-dibromopropyl) phosphate, trimetyl phosphate, tri-o-cresyl phosphate, and tris(methylphenyl) phosphate [149].

Much attention is being paid to flame retardants derived from natural sources, as they are usually non-toxic and environmentally friendly. However, there is much more research for epoxy than unsaturated polyester resins. Good examples are derivatives of gallic acid and 3,4-dihydroxybenzoic acid with a relatively high content of phosphorus. Howell et al. have shown that tris-DOPO phosphonate and the tris-(diethylphosphate) added into epoxy resin do not significantly improve thermal stability. However, the peak heat release rate is considerably reduced. Phosphorus esters of methyl 3,4-dihydroxybenzoate are likewise not much impacted. The peak heat release rate is significantly decreased for the bis-diethylphosphato ester. It was shown that phosphonates better inhibit flammability than corresponding phosphates [155]. Another bio-based flame retardant is tannic acid, which may be used as a hardener for epoxy resin [156], or as an impregnant for carbon fibers when mixed with epoxy monomer [157]. Research shows that the addition of tannic acid results in reducing flammability and smoke generation.

Although nitrogen flame retardants show lower toxicity than other severe types of flame retardants, and although, when under combustion, they do not release any dioxin or halogen acid by-products, there is a concern about their usage [69]. According to REACH, melamine is suspected of damaging fertility or unborn children. Currently, it is also under assessment as persistent, bioaccumulative, and toxic [70]. 

Representatives of boron flame retardants are zinc borate, boric acid, and salts of tetrafluoroboric acid. They do not release toxic gases during decomposition and have low volatility [158]. Additionally, they have a high resistance to harmful biological agents [159]. However, boron present in excessive concentrations becomes toxic. It may inhibit photosynthesis and damage plant cell membranes, resulting in increased permeability [160]. Unfortunately, there is a narrow range of appropriate boron concentration, and it is easy to transcend it. Boron can also accumulate in some organisms, but there is no evidence of biomagnification [161]. 

However, borates, including boric acid and diboron trioxide, are classified as toxic to reproduction for both their developmental and fertility effects, according to Classification, Labelling and Packaging (CLP) regulation. Most of the borates are identified as substances of very high concern, and sodium salts are on the candidate list [162]. In addition, boric acid and its derivatives such as zinc borate, boron zinc oxide, and diboron trioxide are prohibited by the BlueSign^®^ requirements [163]. According to the BlueSign^®^ criteria, manufacturers must act responsibly and sustainably in regard to people, the environment, and resources. The BlueSign^®^ label stands for products manufactured responsibly, with the lowest impact on people and the environment [164].

Tin–zinc compounds, which are substitutes for antimony trioxide, are also considered to be environmentally benign and nontoxic [165]. They also have a low bioaccumulation potential [166].

Commonly used nanoclays are also environmentally safe. Layered silicates can form easily recyclable composites with biodegradable polymers [167]. Carbon materials such as graphene and/or nanotubes are also nontoxic and have no negative environmental impacts. However, they must be used with other flame retardants due to their low effectiveness [168]. POSS combine the characteristics of silica and siloxanes. They show low toxicity and their chemical and thermal stability is high [65].

The environmental impact of intumescent flame retardant systems is dependent on the particular compounds they contain. These coatings are characterized by low toxicity and high efficiency in flame retardancy [169]. It has been shown that coatings made with ammonium polyphosphate, expandable graphite, melamine, and zinc borate consist of graphite, boron trioxide, and boron phosphate, which are thermally stable. During charring processes, no toxic gases are released into the environment [170].

POSS are considered to be non-toxic and environmentally safe flame retardants [171]. Decomposition studies of POSS have shown their low toxicity levels [172].

The use of orthophthalic anhydride in UPR was first suggested to reduce or avoid problems with crystallisation. Resins prepared with orthophthalic anhydride are clear and have good compatibility with styrene. It is a relatively cheap anhydride and is readily available. The isophthalic acid gives tougher cured resins with improved long-term water resistance and lower volatile loss on heating than resins prepared from orthophthalic anhydride. Isophthalic polyesters are also used where some improvement in chemical resistance is required [173]. The UP resins’ properties are very dependent on styrene content. The phase segregation is governed by the crosslink density and by the immiscibility of UP and polystyrene, which depend strongly on the styrene content in the resin. The thermal stability and the mechanical properties of the UP resin reflect the extension of phase segregation [174]. As styrene concentration is increased, it was observed that the gel time increased linearly, the water absorption decreased, the loss modulus “E” decreased, and a higher Tg was obtained at between 5 and 10% [175].

All of the information about the groups of flame retardants used for glass/polyester laminates mentioned are summarized in Table 3. Moreover, the data from the cone calorimeter, smoke density chamber, and limiting oxygen index for the modified and unmodified unsaturated polyester resins or the glass-fiber reinforced composites are collected in Table 4.

## 4. Conclusions

In this review article, various types of flame retardants and their modes of action were considered in the fire retardant application of glass/polyester laminates, and some light was shed on the environmental impact of these compounds. 

Glass/polyester laminates pose a significant fire hazard. Thus, their high flammability has to be reduced in order to obtain an adequate level of fire protection. Diversity of flame retardants enables their use in different industry fields with greater or lesser fire safety requirements.

It has be seen that the flame inhibition strategies are mostly based on the forming of protective thermal barrier layers. However, capturing free radicals by some halogenated FRs, phosphorus FRs, or some organoboron FRs, as well as endothermic dehydration by inorganic hydroxides and nitrogen FRs, was also observed. 

Although traditional flame retardants are commonly applied, there is a growing interest in nanoclays, multi-ingredient intumescent systems, or polyhedral oligomeric silsesquioxanes. Other prospective compounds may be bio-derivatives, which are the subject of research for epoxy resin, mostly as of this moment.

Regarding what is commercially available, there are FRs such as inorganic hydroxides: aluminum trihydroxide and magnesium hydroxide; phosphorus FRs: red phosphorus, ammonium dihydrogen phosphate, and aluminum diethyl phosphinate; nitrogen FRs: melamine and melamine cyanurate; phosphorus–nitrogen FRs: ammonium polyphosphate and melamine polyphosphate; boron FRs: melamine borate; tin and zinc FRs: tin zinc oxide, tin zinc hydroxide, and zinc borate; and intumescent systems containing ammonium polyphosphate. Nanoclays and POSS are not commercially available yet.

Regarding flame retardants considered in improving the fire performance of glass/polyester laminates, besides their distinct effectiveness, in some cases, may adversely affect mechanical properties, especially when higher loadings are required. Incompatibility with the polymer matrix and, consequently, problems with good dispersion as it occurs, may also appear. However, a decrease in the level of flame retardance and the enhancement of the compatibility can be achieved by, for example, the synergism effect or surface modification.

The choice of an appropriate flame retardant should be determined by its fire performance and effectiveness, emerging mechanical properties of the composite, as well as processing methods and price. Another important issue is the environmental impact, the influence on human health, and its ease of recycling. 

## Figures and Tables

**Table 1:** Types of glass fiber reinforcement and their influence on physical properties [15].

Type of Glass Fiber	Enhancement of Properties
S-2 glass	stability, strength, modulus
S glass	tensile strength
R glass	strength, resistance to acid corrosion
AR glass	alkali resistance
A glass	durability, strength, electric resistivity
C glass	resistance to corrosion
D glass	low dielectric constant
E glass	strength, electrical resistivity

**Table 2:** Compositions of intumescent paints and UPR/IFR composite [115,116,117,118].

Number	Component	Content [mass/g]
1	Unsaturated polyester resin	40.0
	Triphenyl phosphate	28.8
	Ammonium polyphosphate	28.8
	Methyl ethyl ketone peroxide	1.6
	Cobalt naphthenate	0.8
2	Unsaturated polyester resin	25.0
	Styrene	0.5
	Ammonium polyphosphate	28.0
	Melamine	17.5
	Methyl ethyl ketone peroxide	0.5
	Cobalt naphthenate	0.04
	Epoxy resin	6.25
	Pentaerythritol	10.5
	Fillers and solvents	11.71
3	Poly(vinyl acetate)	14.4
	Ammonium polyphosphate	39
	Melamine	19.5
	Dipentaerythritol	6.5
	Poly(ethylene terephthalate)	6.5
	Additives	14.1
4	Unsaturated polyester resin	75
	Ammonium polyphosphate	15
	Melamine	5
	Pentaerythritol	5

**Table 3:** A comparison of flame retardants (FR) for glass/polyester laminates.

Type of FR	Examples of Compounds	Mode of Action	Advantages	Disadvantages
Halogenated FRs	Brominated FRs (BFRs): Tetrabromophthalic anhydride, Derivatives of phthalic acid [34], Brominated bisphenols, Polybrominated diphenyl ethers: decabromobiphenyl, 1,2-bis(pentabromophenyl, Derivatives of tetrabromobisphenol acid: tetrabromophthalate diols and polyethers, Derivatives of tetrabromobisphenol A (TBBPA): TBBPA-carbonate oligomer; Poly-di and tribromostyrene, Hexabromocyclododecane [35,37], Chlorinated FRs (CFRs): Tetrachlorophthalic anhydride, Chlorendic acid and its anhydride [34], Polichlorinated bisphenols, Polichlorinated alkanes [36], Chlorinated paraffins [37] Halogen containing phosphorus FRs: tris(2-chloroethyl)phosphate, tris(chloropropyl)phosphate (TClPP), tris(1,3-dichloro-2-propyl)phosphate (TDCPP), tetrekis(2-chloroethyl)dichloroisopentyldiphosphate).	All the halogenated FRs are active in the gas phase through capturing free radicals [37]. In the presence of phosphorus, both the halogen and phosphorus act independently and thus additively [39].	High effectiveness in combustion inhibition, Efficiency in capturing free radicals, Good miscibility and processability, Low price [37,40]. The use of BFRs helps maintain good impact and tensile strength [54].	Enhancement of dense, black smoke emission [32], Not fully biodegradable, Hazardous to the environment and living organisms [40], Tendency to be bioaccumulative, toxic, persistent in the environment. May undergo photodegradation to more toxic compounds [135]. BFRs have low resistance to ultraviolet [54].
Inorganic hydroxides	Aluminum trihydroxide, Magnesium hydroxide [45].	Endothermic dehydration-releasing water molecules decrease temperature. Released metal oxides form additional heat barrier [45].	Low toxicity [151], Low acidity of combustion products [46], Smoke suppressants, Cheap [176].	Effective when their content is high (up to 55%), which may cause difficulties in processing (extrusion or compounding), and changes in mechanical properties, increase in density, decrease in flexibility [32,50] not recommended for composites with a glass content higher than 30% [51].
Phosphorus FRs	Triphenyl phosphate [55], Red phosphorus, Ammonium dihydrogen phosphate [57], Aluminum diethyl phosphinate [62].	May be active both in the gas phase through capturing free radicals and active in the condensed phase by char formation [62,63]. The inorganic phosphate glass may be formed [62].	High efficiency in the gas phase [62], Smoke suppressants [65].	Exhibit toxic effects to people and the environment. Triphenyl phosphate is suspected to be hazardous to aquatic and terrestrial organisms [152], It is registered as very toxic to aquatic life, and with long-lasting effects [153]. Organophosphate FRs can be easily released into environment by volatilization, abrasion, and dissolution [142].
Nitrogen FRs	Melamine, Melamine cyanurate [71,72].	The endothermic decomposition of melamine or related structures lowers the temperature, reduces the concentration of combustible gases by release of ammonia, and produces thermally stable melamine condensates [71,72].	Environmentally friendly, Less toxic than other FRs, Materials containing nitrogen FRs are suitable for recycling [69].	High content, even up to 65% *w*/*w*, is needed to achieve adequate effectiveness, which may reduce mechanical properties and cause difficulties in processing [74]. Melamine is suspected of damaging fertility or unborn children, for now also under assessment of being persistent, bioaccumulative, and toxic [70].
Phosphorus–nitrogen FRs	Ammonium polyphosphate, Melamine polyphosphate [75], Ammonium dihydrogen phosphate [57].	Active in the condensed phase through formation of a protective layer [75].	Highly efficient due to the synergism effect between phosphorus and nitrogen [75], Not high in content (ca. 30 phr) [77].	Comparatively expensive to other flame retardants [177]. Incompatibility of ammonium polyphosphate and melamine polyphosphate with some resins (e.g., epoxy), leading to phase separation [178].
Boron compounds	Melamine borate, Ammonium pentaborate [81], Boron nitride nanosheets [85,86].	Active in the condensed phase through formation of char layer on the surface of the material, decrease in temperature by releasing water molecules from decomposition of boric acid [81,101]. Some organoboron FRs liberate free radicals and non-combustible gases [79].	Low amount of additive ca. 10% [81], Environmentally friendly, of low mammalian toxicity, of low volatility [88,119], Less toxic than other FRs [87], Economically beneficial and cheap [87,120], Smoke suppressants [179].	Highly effective for halogenated resins [81]. In high concentration, boron is toxic to aquatic and terrestrial organisms [89] and can accumulate in some organisms [161]. Borates including boric acid and diboron trioxide are classified as toxic to reproduction. Most of the borates are identified as substances of very high concern [162].
Tin and zinc FRs	Tin zinc oxide, Tin zinc hydroxide [90], Zinc borate [82].	Tin is active in the gas phase through capturing free radicals and in the condensed phase active as a char promoter [91]. Tin and zinc FRs are active in the condensed phase through a formation of a protective layer reducing heat transport into the materials [46].	Smoke suppressants [46], Environmentally benign, Nontoxic [165], Low bioaccumulation potential [166].	Expensive [98], Tendency to aggregate and may be incompatible with polymer matrix [97].
Nanoclays	Montmorillonite and its derivatives [103].	Active in the condensed phase through formation of char layer blocking heat transport to the inside of the material and capturing free radicals. Inorganic nanoclays are active also in the gas phase through capturing free radicals [25].	Highly effective, Environmentally safe [103], Positively influence mechanical properties [104], Low amount of additive (a few percent) [25].	Problems with reaching good dispersion may occur [106].
Intumescent FRs	Intumescent mats and intumescent systems containing three types of compounds: an acid source, e.g., phosphoric acid, boric acid, and their derivatives; a carbonizing source, e.g., polyhydric alcohols, carbohydrates or poly(carbohydrates); a blowing source, e.g., melamine [113].	Active in the condensed phase through formation of porous char layer on the surface, being the thermal barrier reducing heat transport to the inside of the material [46,111,113].	Highly effective, Toxicity depends on the components used, but is usually low [169].	Multi-component [113], migration to the polymer surface may occur due to their low mass [122].
Polyhedral oligomeric silsesquioxanes	POSS derivatives: POSS-graphene oxide [127], POSS-montmorillonite [134], Bentonites modified with POSS [180].	Active in the condensed phase through formation of ceramic layer, being the thermal barrier containing Si–O bonds reducing heat and oxygen transport to the inside of the material. The mode of action may change according to POSS substituents [131].	Effectively enhance thermal stability, Low loads of modifier are required, Improves tensile strength and electric properties [127,134], Environmentally friendly [171], Low toxicity [172].	Problems with aggregation may occur when POSS substituents are not compatible with polymer matrix [131].

**Table 4:** A comparison of flammability and smoke properties of flame retardant glass-fiber reinforced composites with UPR matrix and UPR itself.

Method	Cone Calorimeter (Irradiance Level 50 kW/m^2^)							Smoke Density Chamber	Limiting Oxygen Index	Ref.
Material	Time to Ignition [s]	Peak Heat Release Rate [kW/m^2^]	Time to PHRR [s]	Total Heat Release [MJ/m^2^]	MAHRE [kW/m^2^]	Residual Char Yield [%]	Total Smoke Released [m^2^/m^2^]	Smoke Optical Density D_s max_ [-]	LOI [% *v*/*v*]	
Glass-fiber reinforced UPR	21	328	55	36	-	53	1799	-	-	[123]
Glass-fiber reinforced UPR protected by intumescent mats	5	107	55	48	-	52	963	-	-	
Glass-fiber reinforced UPR	-	508	-	-	-	-	-	-	-	[49]
Organoclay glass-fiber reinforced UPR	-	334	-	-	-	-	-	-	-	
Glass-fiber reinforced UPR	29	343	79	52	-	54	-	-	-	[124]
Glass-fiber reinforced UPR with melamine phosphate	28	262	57	36	-	59	-	-	-	
Glass-fiber reinforced UPR with melamine phosphate and intumescent mat	200	196	385	38	-	63	-	-	-	
Glass-fiber reinforced UPR with melamine pyrophosphate	24	303	57	41	-	59	-	-	-	
Glass-fiber reinforced UPR with melamine pyrophosphate and intumescent mat	213	210	410	46	-	59	-	-	-	
Glass-fiber reinforced UPR with ammonium polyphosphate	23	268	64	37	-	63	-	-	-	
Glass-fiber reinforced UPR with ammonium polyphosphate and intumescent mat	230	175	460	49	-	64	-	-	-	
Glass-fiber reinforced UPR with aluminum trihydrate	30	243	69	45	-	60	-	-	-	
Glass-fiber reinforced UPR with aluminum trihydrate and intumescent mat	251	196	500	42	-	64	-	-	-	
UPR	50 *	792	-	159	459	9	5982	1068 **	21	[119]
UPR with melamine cyanurate	59 *	659	-	144	398	6	4063	773 **	23	
UPR with high nitrogen compound Zn3AT	60 *	419	-	98	247	19	4923	849 **	23	
UPR with high nitrogen compound CUMP	55 *	502	-	111	327	16	4158	844 **	24	
UPR	45	606	-	102	-	0.02	11,272	-	19	[181]
UPR with intumescent flame retardant system	37	259	-	87	-	19	7746	-	28	
UPR with intumescent flame retardant system and montmorillonite	32	213	-	89	-	17	10,833	-	28	
UPR with intumescent flame retardant system and montmorillonite-containing phytic acid	30	223	-	82	-	19	9846	-	29	
UPR	21	870	200	142	382	-	5763	-	-	[76]
UPR with melamine polyphosphate and ammonium polyphosphate	58	146	430	102	101	-	1142	-	-	
UPR with melamine polyphosphate, ammonium polyphosphate and synthetic silica	39	121	600	158	91	-	1610	-	-	
UPR with melamine polyphosphate and aluminium trihydrate	59	166	170	144	101	-	1876	-	-	
UPR with melamine polyphosphate, aluminium trihydrate and synthetic silica	50	184	90	123	117	-	1625	-	-	
UPR with melamine polyphosphate and expandable graphite	30	180	570	162	104	-	998	-	-	
UPR with melamine polyphosphate, expandable graphite and synthetic silica	35	150	770	183	106	-	1747	-	-	
UPR	24	825	-	131	-	-	-	-	-	[52]
UPR with aluminium trihydrate (4.5 m^2^ g^−1^)	55	337	-	122	-	-	-	-	-	
UPR with aluminium trihydrate (4.5 m^2^ g^−1^) and aluminium hypophosphite	58	254	-	92	-	-	-	-	-	
UPR with aluminium trihydrate (4.5 m^2^ g^−1^) and zinc diethylphosphinate	57	265	-	95	-	-	-	-	-	
UPR with aluminium trihydrate (4.5 m^2^ g^−1^) and bis(diphenyl phosphate)	50	241	-	93	-	-	-	-	-	
UPR with aluminium trihydrate (300 m^2^ g^−1^)	61	270	-	104	-	-	-	-	-	
UPR with aluminium trihydrate (300 m^2^ g^−1^) and aluminium hypophosphite	66	248	-	104	-	-	-	-	-	
UPR with aluminium trihydrate (300 m^2^ g^−1^) and zinc diethylphosphinate	56	265	-	96	-	-	-	-	-	
UPR with aluminium trihydrate (300 m^2^ g^−1^) and bis(diphenyl phosphate)	53	250	-	104	-	-	-	-	-	
UPR	34	1153	-	78 (at 240 s)	-	-	3803 (at 90 s)	1320 ***	-	[24]
UPR with ammonium polyphosphate	31	456	-	50 (at 240 s)	-	-	3771 (at 90 s)	422 ***	-	
UPR with ammonium polyphosphate and zinc borate	37	404	-	60 (at 240 s)	-	-	3651 (at 90 s)	525 ***	-	
UPR with ammonium polyphosphate and zinc stannate	28	578	-	60 (at 240 s)	-	-	3048 (at 90 s)	582 ***	-	
UPR with ammonium polyphosphate and zinc hydroxy stannate	35	615	-	67 (at 240 s)	-	-	3743 (at 90 s)	460 ***	-	
UPR with ammonium polyphosphate and montmorillonite	34	453	-	60 (at 240 s)	-	-	3819 (at 90 s)	-	-	
UPR with ammonium polyphosphate, zinc borate and montmorillonite	35	531	-	66 (at 240 s)	-	-	3603 (at 90 s)	444 ***	-	
UPR with ammonium polyphosphate, zinc stannate and montmorillonite	35	586	-	68 (at 240 s)	-	-	3892 (at 90 s)	-	-	
UPR with ammonium polyphosphate, zinc hydroxy stannate and montmorillonite	38	521	-	67 (at 240 s)	-	-	3586 (at 90 s)	-	-	
UPR	44 *	821	-	198	-	516	-	-	-	[25]
UPR with carbon nanotubes	33 *	398	-	198	-	322	-	-	-	
UPR with polyhedral oligomeric silsesquioxane	38 *	464	-	207	-	361	-	-	-	
UPR with titanium dioxide	49 *	531	-	184	-	392	-	-	-	

* measurement at 35 kW/m^2^ irradiance level. ** measurement at 25 kW/m^2^ irradiance level. *** measurement at 50 kW/m^2^ irradiance level.

## Data Availability

The data presented in this study are available on request from the corresponding author.
